# Elderly Patients With Aplastic Anemia: Treatment Patterns and Outcomes in the Real World

**DOI:** 10.1002/ajh.27611

**Published:** 2025-01-29

**Authors:** Bruno Fattizzo, Carmelo Gurnari, Sabrina Giammarco, Antony Ricchiuti, Hussein Awada, Marta Bortolotti, Nicole Galli, Giacinto Luca Pedone, Francesco Versino, Dario Consonni, Roochi Trikha, Shreyans Gandhi, Simona Sica, Jaroslaw P. Maciejewski, Austin Kulasekararaj, Wilma Barcellini

**Affiliations:** ^1^ Department of Oncology and Hemato‐Oncology University of Milan Milan Italy; ^2^ Fondazione IRCCS Ca' Granda Ospedale Maggiore Policlinico Milan Italy; ^3^ Department of Translational Hematology and Oncology Research Taussig Cancer Institute, Cleveland Clinic Cleveland Ohio USA; ^4^ Department of Biomedicine and Prevention University of Rome Tor Vergata Rome Italy; ^5^ Fondazione Policlinico Universitario A. Gemelli‐Istituto di Ricovero e Cura a Carattere Scientifico (IRCCS) Rome Italy; ^6^ Hematology Unit, King's College Hospital London UK

**Keywords:** anti‐thymocyte globulin, aplastic anemia, cyclosporine, elderly, eltrombopag

## Abstract

We retrospectively analyzed a large international cohort of 1113 patients with aplastic anemia to evaluate treatment choice and outcome in elderly patients as compared with a younger population. Overall, 319 (29%) patients were > 60 years old at diagnosis (60–64 years (*n* = 85), 106 65–69 years (*n* = 106), and 128 > 70 years (*n* = 128)). Elderly patients showed a more severe thrombocytopenia at onset and a significantly lower overall response (complete plus partial) to first‐line therapy at 6 months as compared to younger patients (47% vs. 65%, *p* < 0.0001), irrespective of treatment modality (ATG or CyA combinations, eltrombopag, or androgens); 27 (8%) received transplant as second line therapy and 11 (41%) died, mainly due to transplant complications. The rate of evolution to MDS was greater in elderly patients (12% vs. 7% in younger AA, *p* = 0.002), whilst the rate of evolution to AML was similar (1.8 vs. 1.3%). By multivariable analysis, older age remained the main factor associated with mortality [HR 1.64 (95% CI 1.5–1.7), *p* < 0.001], followed by disease severity by Camitta classification [HR 2.24 (95% CI 1.6–3.1) for severe AA; HR 3.8 (95% CI 2.4–6) for very severe AA], and male gender [1.45 (95% CI 1.1–1.8), *p* < 0.001]. In this large study, elderly AA was associated with inferior outcome even in the TPO‐RA era, highlighting the need for further optimization of clinical management.

## 1 Introduction

Aplastic anemia (AA) is a rare bone marrow failure disorder characterized by a bimodal incidence with peaks in the pediatric‐young adults and in the elderly > 60 years [1]. The latter patient population may be particularly frail due to comorbidities that may also impede tolerability of standard treatment. Typically, patients are treated with anti‐thymocyte globulin (ATG) and cyclosporine with eltrombopag based on the results of the Phase 3 randomized controlled trial [2, 3]. However, because of age‐related comorbidities, many subjects are considered not candidate to full dose immunosuppressive therapy (IST), and are treated with either cyclosporine alone, androgens, or eltrombopag monotherapy [4]. Furthermore, the outcome of treatment with immunosuppressive therapy and allogeneic hematopoietic cell transplant (HCT) has been clearly shown to be inferior in the elderly [2, 3, 4, 5].

To date, little evidence is available since the introduction of novel treatment modalities such as eltrombopag. Here, we retrospectively analyzed a large international cohort of patients with aplastic anemia to evaluate treatment choice and outcome in elderly patients as compared with a younger population to inform future treatment strategies in this setting.

## 2 Patients and Methods

We accrued patients diagnosed with AA according to Camitta criteria at four tertiary hematologic centers in Italy (Milan and Rome), USA, and UK between 1976 and 2024. The study was conducted according to the Helsinki declaration and patients gave informed consent. Details on baseline hematologic features, bone marrow evaluation, and mutational status by next‐generation sequencing (NGS) of common myeloid drivers were reviewed. The different management strategies were registered and categorized as: (1) cyclosporin single agent (CyA), (2) eltrombopag + CyA, (3) eltrombopag single agent, (4) ATG combinations, (5) androgens, and (6) HCT. Responses were assessed according to EBMT criteria (complete, CR, if platelets PLT > 100 × 10^9^/L, hemoglobin Hb > 10 g/dL, neutrophils‐ANC > 1.5 × 10^9^/L; partial, PR if transfusion independence).

All adverse events (AEs), including infectious, thrombotic, and bleeding complications, were graded according to the Common terminology criteria CTCAE version 5. The occurrence of evolution into hemolytic PNH and myeloid neoplasms (namely MDS or AML), as well as death and the relative causes were also recorded for all patients.

## 3 Statistical Analysis

Kruskal–Wallis and chi‐squared tests were used for comparison of quantitative and categorical variables, respectively. Survival analysis was performed after truncation of follow‐up time (time since diagnosis to last follow‐up) at 15 years. Overall survival (OS) and mortality was analyzed using the Kaplan–Meier estimator. Hazard ratios (HR) and 95% confidence intervals (CI) were calculated for selected variables using univariate ad multivariable Cox regression models. We graphed cumulative incidence curves for myeloid neoplasms and PNH; death and each of these two conditions were treated as reciprocal competing events. Statistical analyses were performed with Stata 17 (StataCorp. 2021).

All data were analyzed according to age categories < 60 vs. ≥ 60 years (namely young vs. elderly AA); the latter were further divided into 60–64 vs. 65–69 vs. ≥ 70 years categories for continuous variables analyses. Patients gave informed consent, and the study was conducted according to guidelines set forth by the Helsinki Declaration.

## 4 Results

Overall, 1113 patients were included, with a median age of 45 years (range 22–78) and a male to female ratio of 1:1. According to age at presentation, 319 (29%) patients were ≥ 60 years old (*N* = 85 were 60–64 years, 106 65–69 years, and 128 ≥ 70 years category) and 794 (71%) < 60 years old at diagnosis. As shown in Table 1, distribution of different severity categories was comparable across ages (overall, 44% non‐severe AA, 46% severe AA, and 10% very severe AA). The severity of thrombocytopenia at presentation was greater in elderly vs. younger patients (*p* = 0.001), whilst mean Hb and neutrophil levels were comparable. Similarly, the rate of red cell and platelet transfusions (79% of cases in < 60 vs. 81% in ≥ 60 years and 78% vs. 85%, respectively in 60–64 and 65–69 groups) were not significantly different between elderly and younger patients. A PNH clone was detected in 43% of patients with similar frequency across ages, but with a smaller size on granulocytes in elderly patients (*p* = 0.002). Furthermore, elderly patients more frequently presented cytogenetics aberrations (11% vs. 5%, *p* = 0.004), none MDS defining, whilst the frequency of somatic mutations by NGS myeloid panel (21% overall) was comparable among age subgroups (Table S1). Bone marrow reticulin fibrosis (WHO grade 1), as observed in AA, was present in 14% of patients, without differences across ages.

**TABLE 1:** Clinical and hematological features of aplastic anemia (AA) patients according to age.

	< 60 years	≥ 60 years	60–64 years	65–69 years	≥ 70
	*N* = 794	*N* = 319	*N* = 85	*N* = 106	*N* = 128
Age, years—median (range)	34 (22–48)	68 (65–78)	62.6 (61.4–63.7)	67.6 (66.5–68.5)	74.3 (72.1–78.1)
Males/females	396/398	162/157	43/42	54/52	65/63
AA category, *N* (%)					
Moderate	350 (44%)	137 (43%)	40 (47%)	44 (42%)	53 (41%)
Severe	360 (45%)	155 (49%)	36 (42%)	50 (47%)	69 (54%)
Very severe	84 (11%)	27 (8%)	9 (11%)	12 (11%)	6 (5%)
PLT × 10^9^/L—median (range)	26 (14–52)	22 (11–40)*	26 (15–46)	24 (14–40)	18.5 (10–32)
Hb g/dL—median (range)	9.2 (7.9–10.5)	9.3 (8.1–10.4)	9.0 (8–10.6)	9.3 (8.4–10.2)	9.3 (8–10.3)
ANC × 10^9^/L—median (range)	0.8 (0.3–1.4)	0.8 (0.3–1.5)	1.0 (0.3–1.7)	0.8 (0.3–1.7)	0.7 (0.3–1.4)
LDH U/L–median (range)	198 (163–260)	219 (177–259)	231 (185–265)	204 (157–254)	220 (184–259)
Ret × 10^9^/L—median (range)	38 (16–61)	33 (16–58)	34 (21–62)	24 (10–58)	38 (18–53)
Endogenous EPO U/L—median (range)	551 (232–1052)	469 (167–760)	434 (153–815)	453 (257–623)	478 (164–889)
RBC transfusions, *N* (%)	417/527 (79%)	167/205 (81%)	36/46 (78%)	62/73 (85%)	69/86 (80%)
PLT transfusions, *N* (%)	401/527 (76%)	168/202 (83%)	36/45 (80%)	62/73 (85%)	70/84 (83%)
BM cellularity %—median (range)	10 (5–20)	10 (5–15)	10 (5–19)	10 (5–16)	10 (5–10)
BM fibrosis WHO grade 1, *N* (%)	33/256 (13%)	23/134 (17%)	4/32 (13%)	6/43 (14%)	13/59 (22%)
Cytogenetics abnormalities, *N* (%)	35/692 (5%)	29/274 (11%)	10/72 (14%)	11/94 (12%)	8/108 (7%)
NGS abnormalities, *N* (%)	58/297 (20%)	29/152 (19%)	1/34 (3%)	16/57 (28%)	12/61 (20%)
PNH clone positivity, *N* (%)	348/731 (48%)	131/294 (45%)	37/78 (47%)	46/101 (46%)	48/115 (42%)
Granulocyte PNH clone size %–median (range)	2.5 (0.3–20)	1.2 (0.3–5.3)**	1.0 (0.2–4.6)	1 (0.2–4.0)	1,2 (0.4–4.5)

* *p* = 0.001.

** *p* = 0.002.

Abbreviations: ANC, absolute neutrophil counts; BM, bone marrow; EPO, erythropoietin; Hb, hemoglobin; LDH, lactate dehydrogenase; NGS, next generation sequencing; PLT, platelets; PNH, paroxysmal nocturnal hemoglobinuria; Ret, reticulocytes.

Median time from diagnosis to first‐line treatment was similar among elderly and younger patients: 1.6 months in younger (0–80) and 1.5 months in elderly patients (0–78) (Table 2). ATG plus CyA was administered in 39% of patients aged ≥ 60 years and 52% of those younger than 60 years, ATG plus CyA and eltrombopag in 3% and 5%, CyA only in 30% and 14%, respectively (*p* < 0.0001), CyA plus eltrombopag in 7% and 3%, eltrombopag only in 4% and 1%, and androgens in 1.9 and 2%, respectively. Elderly patients showed a significantly lower overall response (complete plus partial) to first‐line therapy at 6 months as compared to younger patients (47% vs. 65%, *p* < 0.0001), irrespective of treatment modality. Regarding response to specific treatments, elderly patients showed a significant lower response to ATG plus CyA with or without eltrombopag as compared to younger subjects (39% vs. 62%, *p* < 0.0001), whilst cyclosporine, eltrombopag and their combination yielded responses in about half of cases, regardless age. Finally, 4% of patients aged ≥ 60 years and 8% of < 60 years received no treatment.

**TABLE 2:** Treatment modalities in aplastic anemia patients according to age category.

First‐line approach	< 60 years	≥ 60 years	60–64 years	65–69 years	≥ 70
	*N* = 794	*N* = 319	*N* = 85	*N* = 106	*N* = 128
Untreated	62 (8)	14 (4)	4 (5)	4 (4)	6 (5)
Cyclosporine alone	114 (14)	96 (30)*	21 (25)	28 (26)	47 (37)
Response	60 (53)	42 (44)	10 (48)	11 (39)	21 (45)
AE	18 (16)	11 (11)	3 (14)	3 (11)	5 (11)
Cyclosporine plus eltrombopag	24 (3)	22 (7)	2 (2)	8 (8)	12 (9)
Response	16 (67)	12 (54)	1 (50)	5 (63)	6 (50)
AE	1 (4)	4 (18)	1 (50)	2 (25)	1 (8)
Eltrombopag alone	6 (1)	12 (4)	3 (4)	3 (3)	6 (5)
Response	4 (67)	6 (50)	0	2 (67)	4 (67)
AE	0	0	0	0	0
ATG plus cyclosporine	405 (52)	126 (39)	46 (54)	47 (44)	33 (26)
Response	258 (64)	50 (39)*	20 (43)	17 (36)	13 (39)
AE	41 (10)	15 (12)	5 (11)	6 (13)	4 (12)
ATG plus cyclosporine plus eltrombopag	37 (5)	10 (3)	4 (5)	3 (3)	3 (2)
Response	17 (46)	3 (30)	2 (50)	1 (33)	0 (0)
AE	3 (8)	2 (20)	0 (0)	1 (33)	1 (30)
Androgens	17 (2)	6 (2)	0 (0)	4 (3)	2 (1.5)
Response	9 (53)	4 (67)		2 (67)	2 (100)
AE	0	1 (17)		1 (33)	0
Transplant					
1st line, *N* (%)	70 (9)	0	0	0	0
2nd line, *N* (%)	152 (19)	27 (8)	15 (18)	11 (10)	1 (1)
Further line, *N* (%)	4 (0.5)	0	0	0	0
Eculizumab, *N* (%)	90 (11%)	19 (6%)	5 (6%)	5 (5%)	9 (7%)
Death, *N* (%)	133 (17%)	154 (48%)	25 (29%)	49 (46%)	80 (63%)
MDS evolution, *N* (%)	53 (7%)	39 (12%)	14 (16%)	14 (13%)	11 (9%)
AML evolution, *N* (%)	14 (2%)	4 (1%)	0 (0%)	2 (2%)	2 (2%)
PNH evolution, *N* (%)	87 (11%)	19 (6%)	6 (7%)	5 (5%)	8 (6%)

* *p* < 0.01.

Abbreviations: AE, adverse events; AML, acute myeloid leukemia; ATG, anti‐thymocyte globulin; MDS, myelodysplastic neoplasms; PNH, paroxysmal nocturnal hemoglobinuria.

Concerning adverse events, the overall rate (10%) was similar among elderly and younger patients (Table S2), with the most common toxicities being renal failure with cyclosporine combinations (5%), and thrombosis in patients treated with ATG plus CyA (4%).

No elderly patient underwent frontline transplant (0% vs. 9% of patients ≤ 60 years, *p* < 0.001), whilst 27 (8%) received transplant as second line therapy (vs. 19% of younger cases, *p* < 0.0001). Of these 27 elderly transplanted patients (median age 63 years, range 60–71; 3 non severe, 18 severe, and 6 very severe AA), 11 died (41%; 2 non severe, 7 severe, 2 very severe AA) due to post‐transplant infections (*N* = 5), GVHD (*N* = 2), VOD (*N* = 1), metastatic adenocarcinoma of caecum (*N* = 1), and unknown causes (*N* = 2).

Overall, 10% of patients required eculizumab therapy for their expanding PNH clone with hemolysis at diagnosis, less commonly in the elderly subgroup (6% versus 11%, *p* = ns).

**FIGURE 1:**
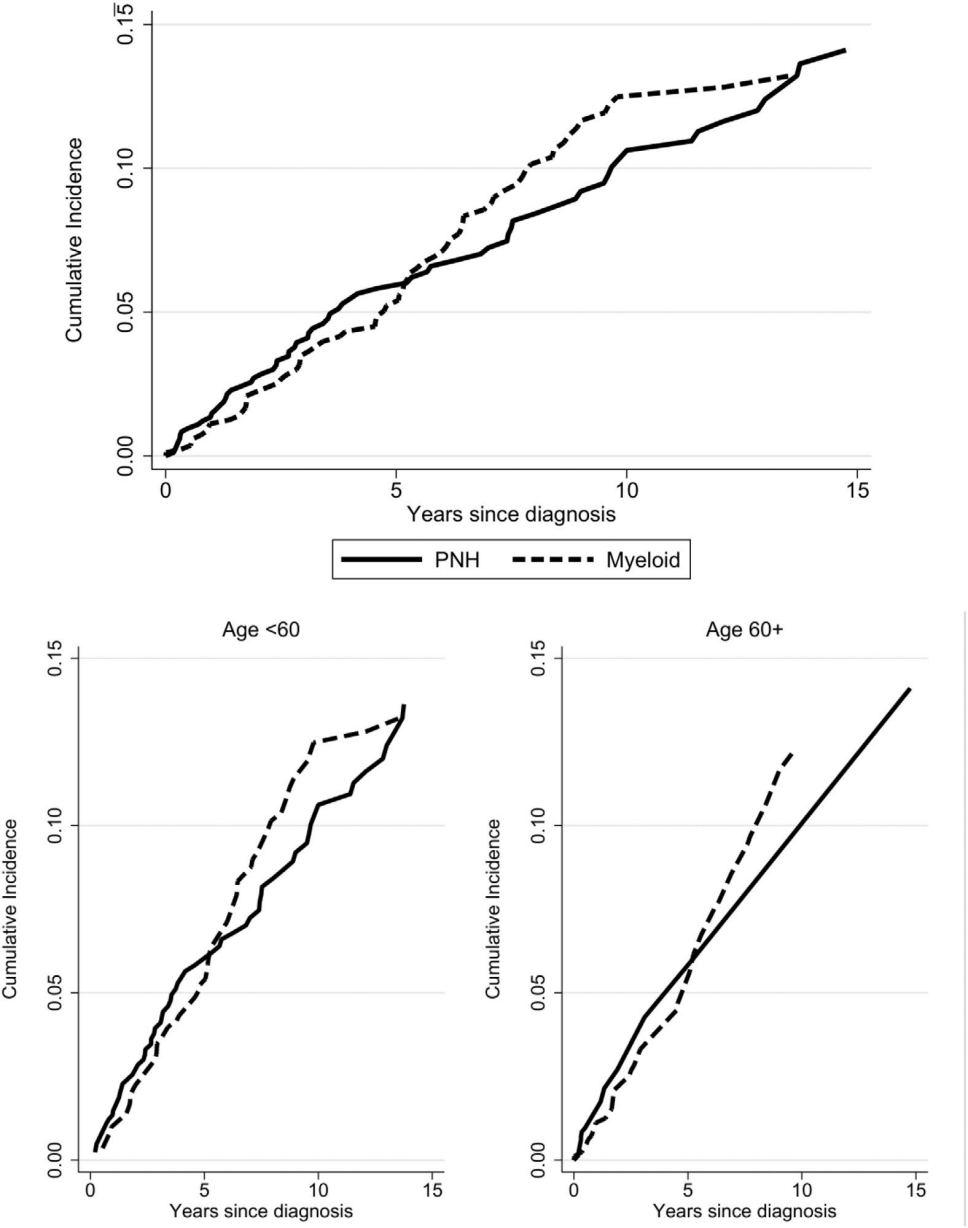
Cumulative incidence of evolution into myeloid neoplasm and paroxysmal nocturnal hemoglobinuria. Survival of aplastic anemia patients according to age category. Upper panel shows cumulative data, and lower panel shows data stratified according to age at diagnosis.

The rate of evolution to MDS was greater in elderly patients (12% vs. 7% in younger AA, *p* = 0.002), whilst the rate of evolution to AML was similar across age groups (1.8 vs. 1.3%). Evolution to MDS/AML was not associated with no response/partial response to therapy (44% in elderly and 30% in younger AA) nor with molecular lesions at NGS (25% in elderly and 11% in younger AA). Furthermore, 6% of elderly and 11% of younger AA patients (*p* = 0.01) developed a clinically significant PNH clone requiring anti‐complement therapy (Figure 1).

**FIGURE 2:**
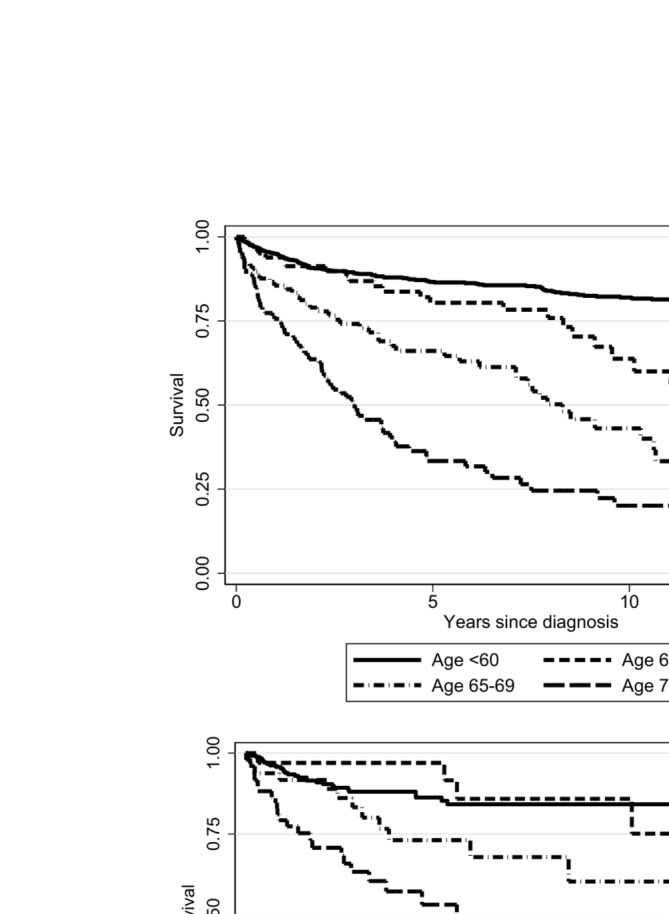
Overall survival of aplastic anemia patients according to age category. Upper panel shows cumulative data over a 15 year follow up; lower panel shows the analysis restricted to those patients treated after 2014.

**FIGURE 3:**
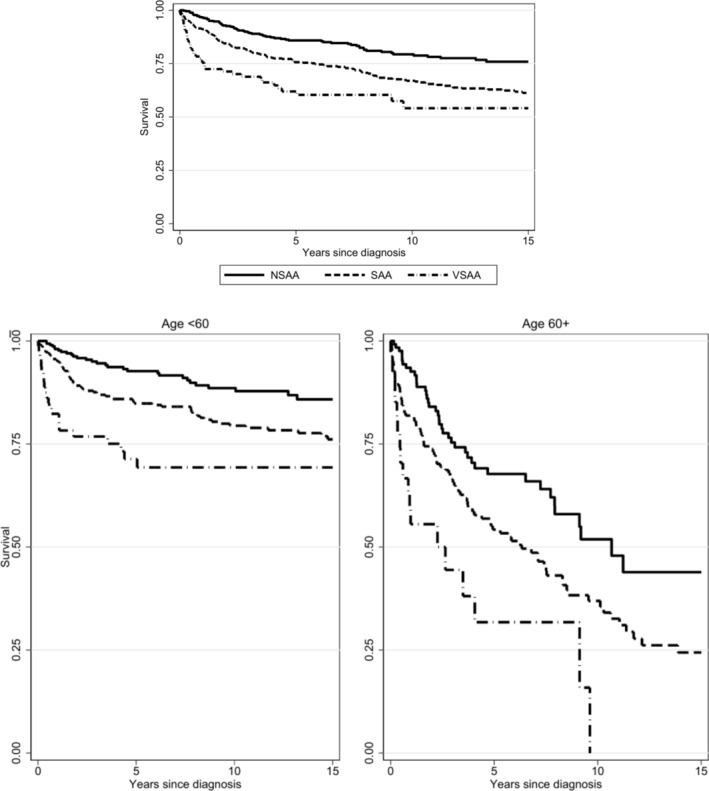
Overall survival of aplastic anemia patients according to Camitta criteria. Upper panel shows cumulative data, and lower panel shows data stratified according to age at diagnosis.

During the follow up time (median 75 months, IQR 67–262) 287 patients (26%) died, with an increasing frequency across age categories (17% in < 60 years, 29% 60–64, 46% 65–69, and 63% ≥ 70 years; Figure 2 upper panel). Infectious complications were the most frequent cause of death independently from treatment strategy (Table S3), followed by evolution to MDS/AML. The following hazard ratios for age‐related mortality emerged as compared to younger patients: 1.97 (95% CI 1.2–3) for 60–64 years, 4 (2.8–5.7) for 65–69 years, and 7.4 (5.5–9.9) for ≥ 70 years. By multivariable analysis, older age remained the main factor associated with mortality [HR 1.64 (95% CI 1.5–1.7)], followed by disease severity by Camitta classification (Figure 3) [HR 2.24 (95% CI 1.6–3.1) for severe AA; HR 3.8 (95% CI 2.4–6) for very severe AA], and male gender [1.45 (95% CI 1.1–1.8)]. No associations with mortality risk and treatment modalities were observed (Figure S1), although patients not requiring treatment due to a less severe disease (mainly moderate/non severe AA) showed the best survival (non‐significant).

By restricting the analysis to patients treated after 2014, when eltrombopag became available (*N* = 381 of whom 111 treated with eltrombopag either alone or in combination) we confirmed that all‐cause mortality was higher in elderly versus younger patients, without association with treatment type (Figure 2, lower panel).

## 5 Discussion

In this large real‐world AA multicenter series, older age was associated with more severe thrombocytopenia at onset, poorer response to first‐line treatment, particularly with ATG combinations, higher rate of transformation to MDS and worse survival. This population constitutes a difficult‐to‐treat setting despite the availability of novel therapeutic modalities, suggesting the need for further optimization of clinical management.

One of the possible reasons for such poor outcome could be the fear of adverse events due to comorbidities, which could possibly lead to undertreat such elderly patients. In our study, despite a similar percentage of patients treated with ATG among elderly and younger subjects, and a similar rate of AEs, response rates and outcome were significantly inferior in the former. Accordingly, the most recent BSH guidelines for AA warrant a cautious use of ATG in patients older than 60 years [1].

Additionally, one could speculate that a proportion of patients might have a misdiagnosed hypoplastic MDS that may contribute to the poor outcome [6]. As a matter of fact, the frequency of cytogenetics aberrations was higher among elderly patients, although none of them was MDS defining as per WHO/ICC [7, 8]. Consistently, the number and type of somatic mutations by NGS were similar in elderly and younger patients and were not associated with outcome.

Even the addition of eltrombopag did not seem to significantly impact response to treatment in elderly patients. This is in line with the recent phase 3, randomized, placebo‐controlled RACE trial. Comparing eltrombopag plus IST with horse ATG and cyclosporine versus IST only, the study showed significantly better outcome in younger patients. Particularly, in the multivariable analysis of the trial, age was a significant factor associated with response [2].

Androgens, aimed at boosting stem cell machinery and telomere elongation [9], did not favorably impact on outcome of elderly AA in this study. However, they may remain a useful tool in those failing or not candidate to IST, and in patients previously exposed to eltrombopag, or where the latter is not available.

To summarize, ATG combinations require patient hospitalization, may imply the risk of serum sickness and anaphylaxis, and induce a profound and long‐lasting immunosuppression with increased risk of opportunistic infections. In our series, while the rate of adverse events was similar across ages, the response rate was dramatically inferior in the elderly (particularly around 30% in those > 65 years old), suggesting a better risk/benefit ratio for outpatient regimens such as cyclosporine with or without eltrombopag in this setting (response rates 50%–60%). Accordingly, patients > 40 years old enrolled in the RACE trial showed a response to ATG plus cyclosporine with or without eltrombopag in 30%–50% of cases [2]. Additionally, responses to cyclosporine plus eltrombopag were favorable in a recent trial exploring ATG free combinations [3]. Furthermore, in a previous experience, the combination of cyclosporine plus eltrombopag was the one to induce the highest rate of trilineage response in non‐severe AA and might therefore be preferred in this patient‐population [10]. Androgens induce a response in 40%–50% of patients, but may be burdened by several adverse events, particularly virilization, liver toxicity, and possible alterations of lipid profile; they may be considered as second/third line of treatment in patients failing cyclosporine and eltromboag. Finally, the very poor outcome of transplant in our elderly cohort seems to defer this option after the second line in patients with severe/very severe AA or with dysplastic evolution.

**FIGURE 4:**
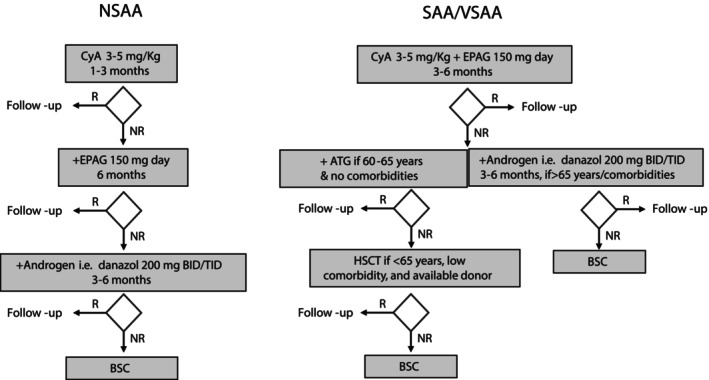
A proposed algorithm for the management of non‐severe, severe/very severe aplastic anemia (NSAA, SAA/VSAA) elderly patients. ATG, anti‐thymocyte globulin; BSC, best supportive care; CYA, cyclosporine A; EPAG, eltrombopag; NR, no response; R, response. [Color figure can be viewed at wileyonlinelibrary.com]

A proposed algorithm has been depicted in Figure 4: for NSAA, CyA can be initiated for 1 to 3 months and addition of eltrombopag considered if no response; after 3–6 months of combined treatment, if no response, androgens can be added provided absence of contraindications. In SAA/VSAA, a frontline combination approach with CyA plus eltrombopag could be considered, with the addition of ATG in patients < 65 years, if no response after 3–6 months, versus the addition of androgens in more elderly patients or those with comorbidities, provided absence of contraindications; if no response after 3–6 months, absence of significant comorbidities and available donor, HSCT could be considered.

Our study carries the limitations of a retrospective analysis. However, the inclusion of a large number of patients with such a rare disease with both severe and non‐severe phenotypes, involving experienced centers, corroborate the data‐driven messages of this report.

In conclusion, these observations suggest an unfavorable risk/benefit ratio for ATG, whilst cyclosporine, eltrombopag or their combination may be the best choice in elderly AA patients although with responses in half of patients only. There is still room for optimizing treatment strategies in elderly AA, for instance by including other immunosuppressive agents such as mTOR inhibitors as well as second generation thrombopoietin receptor agonists as romiplostim and avatrombopag, showing efficacy signals in early reports [11, 12, 13].

## Author Contributions

All authors equally contributed to data collection, literature revision, manuscript writing and editing.

## Conflicts of Interest

The authors declare no conflicts of interest.

## Supporting information


**Data S1.** Supporting Information.

## Data Availability

All data were included in the article manuscript and Supporting Information. Additional information may be obtained upon reasonable request to the corresponding author.
