# Supplementary material for: Elderly Patients With Aplastic Anemia: Treatment Patterns and Outcomes in the Real World
Source: Am J Hematol. 2025 Jan 29;100(4):584–91. doi: 10.1002/ajh.27611 (PMC11886494; doi:10.1002/ajh.27611)
Supplement: Supplementary file 1 — Data S1. Supporting Information. [file AJH-100-584-s001.docx]

**Supplementary Figure 1: overall survival according to treatment strategies according to age.**

Therapy 0 untreated; 1 cyclosporine; 2-3 cyclosporine plus eltrombopag and eltrombopag single agent; 4 androgens; 5 ATG combinations; 6 transplant.


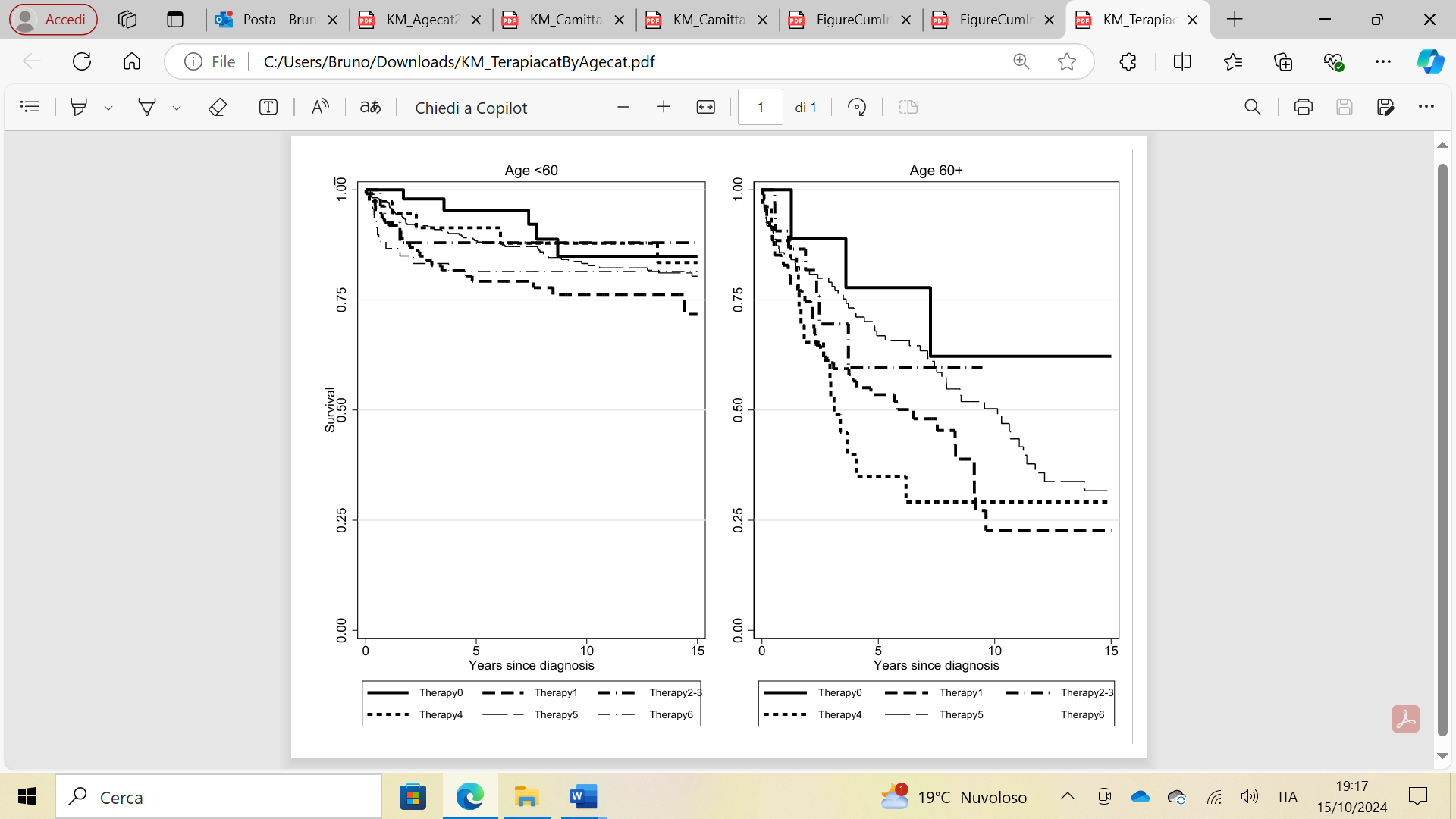


**Supplementary table 1: next generation sequencing results by age category.**

|  | **tested** | **positive** | **%** | **type of mutations, N.** |
| --- | --- | --- | --- | --- |
| <60 years | 297 | 63 | 21 | ASXL1 – 12  CBL - 5  RUNX1 - 5  TET2 – 16  STAG2 – 1  EZH2 – 9  ZRSR2 – 4  SETBP1 – 1  BCOR – 5  CARL – 2  EP300 – 2  CEBPA – 1  DNMT3A – 3  CSF3R – 1  CUX1 – 4  MPL – 1  NF1 – 1  KIT – 1  U2AF1 – 1  JAK2 – 1  FLT3 – 1  PRPF8 – 1  WT1 – 1  SF3B1 – 1  SRSF2 – 1  ETV6 – 1  SBDS – 1 |
| >/= 60 years | 152 | 29 | 19 | ASXL1 – 3  NRAS – 2  CBL - 1  RUNX1 – 3  IKZF1 1 – 1  PHF6 – 1  BCORL –2  TET2 – 5  STAG2 – 1  EZH2 – 1  ZRSR2 – 1  BCOR – 3  DNMT3A – 5  CUX1 – 3  NF1 – 3  KIT – 1  U2AF1 – 2  PRPF8 – 1  SF3B1 – 1  SRSF2 – 1  ETV6 – 1  RAD21 – 1 |

|  | Cyclosporine | RENAL | CARDIO | NEURO | THROMBOSIS | GASTRO | SKIN | ANAPHYLAXIS | CMV REACTIVATION | OSTENECROSIS | INFECTIONS | SERUM SICKNESS | UNSPECIFIED | DEATH |
| --- | --- | --- | --- | --- | --- | --- | --- | --- | --- | --- | --- | --- | --- | --- |
| N=114 | <60 | 2 | 3 | 1 | 0 | 0 | 0 | 0 | 0 | 0 | 0 | 0 | 0 | 25 (22%) |
| N=96 | >60 | 5 | 0 | 1 | 2 | 2 | 0 | 0 | 0 | 0 | 0 | 0 | 1 | 50 (52%) |
|  |  |  |  |  |  |  |  |  |  |  |  |  |  |  |
|  | Cyclosporine + eltrombopag | RENAL | CARDIO | NEURO | THROMBOSIS | GASTRO | SKIN | ANAPHYLAXIS | CMV REACTIVATION | OSTENECROSIS | INFECTIONS | SERUM SICKNESS | UNSPECIFIED | DEATH |
| N=24 | <60 | 0 | 0 | 0 | 0 | 1 | 0 | 0 | 0 | 0 | 0 | 0 | 0 | 2 (8%) |
| N=22 | >60 | 1 | 2 | 0 | 0 | 2 | 0 | 0 | 0 | 0 | 0 | 0 | 0 | 5 (23%) |
|  |  |  |  |  |  |  |  |  |  |  |  |  |  |  |
|  | Androgens | RENAL | CARDIO | NEURO | THROMBOSIS | GASTRO | SKIN | ANAPHYLAXIS | CMV REACTIVATION | OSTENECROSIS | INFECTIONS | SERUM SICKNESS | UNSPECIFIED | DEATH |
| N=17 | <60 | 0 | 0 | 0 | 0 | 0 | 0 | 0 | 0 | 0 | 0 | 0 | 0 | 6 (35%) |
| N=8 | >60 | 0 | 0 | 0 | 1 | 0 | 0 | 0 | 0 | 0 | 0 | 0 | 0 | 4 (50%) |
|  |  |  |  |  |  |  |  |  |  |  |  |  |  |  |
|  | Anti-thymocyte globulin | RENAL | CARDIO | NEURO | THROMBOSIS | GASTRO | SKIN | ANAPHYLAXIS | CMV REACTIVATION | OSTENECROSIS | INFECTIONS | SERUM SICKNESS | UNSPECIFIED | DEATH |
| N=442 | <60 | 7 | 1 | 4 | 20 | 4 | 2 | 1 | 1 | 1 | 1 | 0 | 1 | 70 (16%) |
| N=136 | >60 | 4 | 1 | 2 | 2 | 3 | 2 | 0 | 1 | 0 | 1 | 1 | 0 | 65 (48%) |
|  |  |  |  |  |  |  |  |  |  |  |  |  |  |  |
|  | Eltrombopag | RENAL | CARDIO | NEURO | THROMBOSIS | GASTRO | SKIN | ANAPHYLAXIS | CMV REACTIVATION | OSTENECROSIS | INFECTIONS | SERUM SICKNESS | UNSPECIFIED | DEATH |
| N=6 | <60 | 0 | 0 | 0 | 0 | 0 | 0 | 0 | 0 | 0 | 0 | 0 | 0 | 1 (17%) |
| N=12 | >60 | 0 | 0 | 0 | 0 | 0 | 0 | 0 | 0 | 0 | 0 | 0 | 0 | 3 (25%) |

**Supplementary table 2. Adverse events type and distribution among younger and elderly subjects**

| **N. of deaths** | **Available data** | **Treatment** | **Infections** | **Hematopoietic cell transplant complications** | **Acute myeloid leukemia** | **Other cancer** | **Bleeding** |
| --- | --- | --- | --- | --- | --- | --- | --- |
|  |  | Cyclosporine |  |  |  |  |  |
| 25 | 18 | <60 years | 10 | 5 | 2 | 1 | 0 |
| 50 | 23 | >60 years | 18 | 1 | 3 | 1 | 0 |
|  |  |  |  |  |  |  |  |
|  |  | Cyclosporine plus eltrombopag |  |  |  |  |  |
| 2 | 1 | <60 years | 1 | 0 | 0 | 0 | 0 |
| 5 | 2 | >60 years | 1 | 0 | 0 | 1 | 0 |
|  |  |  |  |  |  |  |  |
|  |  | Androgens |  |  |  |  |  |
| 6 | 6 | <60 years | 5 | 0 | 1 | 0 | 0 |
| 18 | 10 | >60 years | 8 | 0 | 2 | 0 | 0 |
|  |  |  |  |  |  |  |  |
|  |  | Anti-thymocyte globulin |  |  |  |  |  |
| 70 | 37 | <60 years | 28 | 3 | 2 | 2 | 2 |
| 65 | 34 | >60 years | 21 | 1 | 9 | 2 | 1 |
|  |  |  |  |  |  |  |  |
|  |  | Eltrombopag |  |  |  |  |  |
| 1 | 1 | <60 years | 1 | 0 | 0 | 0 | 0 |
| 3 | 3 | >60 years | 2 | 0 | 0 | 1 | 0 |
|  |  |  |  |  |  |  |  |
|  |  | Untreated |  |  |  |  |  |
| 6 | 5 | <60 years | 2 | 0 | 2 | 1 | 0 |
| 2 | 2 | >60 years | 1 | 0 | 1 | 0 | 0 |
|  |  |  |  |  |  |  |  |
|  |  | Hematopoietic cell transplant |  |  |  |  |  |
| 15 | 9 | <60 years | 5 | 3 | 1 | 0 | 0 |
| 0 | 0 | >60 years | 0 | 0 | 0 | 0 | 0 |

**Supplementary table 3. Causes of death in younger and elderly subjects with aplastic anemia according to frontline treatment strategy.**
